# Identification of lncRNA DLEU2 as a potential diagnostic biomarker and anti-inflammatory target for ulcerative colitis

**DOI:** 10.3389/fphar.2022.991448

**Published:** 2022-09-14

**Authors:** Qiuling Lin, Dingguo Zhang, Jian Zhang, Weixiang Luo, Zhenglei Xu, Jun Yao, Lisheng Wang

**Affiliations:** ^1^ Department of General Practice, Shenzhen People’s Hospital (The Second Clinical Medical College, Jinan University; The First Affiliated Hospital, Southern University of Science and Technology), Shenzhen, Guangdong, China; ^2^ Department of Gastroenterology, Shenzhen People’s Hospital (The Second Clinical Medical College, Jinan University; The First Affiliated Hospital, Southern University of Science and Technology), Shenzhen, Guangdong, China; ^3^ Department of Plastic Surgery, Sun Yat-Sen Memorial Hospital, Sun Yat-Sen University, Guangzhou, Guangdong, China; ^4^ Department of Nursing, Shenzhen People’s Hospital (The Second Clinical Medical College, Jinan University; The First Affiliated Hospital, Southern University of Science and Technology), Shenzhen, Guangdong, China

**Keywords:** ulcerative colitis, DLEU2, inflammatory factors, NF-κB, lncRNA

## Abstract

The incidence of ulcerative colitis (UC) in China has significantly increased over the past 10 years. Here we aim to explore potential diagnostic biomarkers and anti-inflammatory targets associated with UC. Patients with UC were enrolled in this study. The expression of lncRNAs and mRNAs in the nidus of the gut mucosa and adjacent normal mucosa samples was evaluated by RNA sequencing. The role of DLEU2 in inflammation and NF-κB signaling pathway was examined by RT-qPCR, Western blotting, and ELISA with human macrophage-like cells derived from THP-1. 564 lncRNAs and 859 mRNAs are significantly altered in the nidus of the gut mucosa of UC patients. Among the differentially expressed lncRNAs, DLEU2 changes the most. The expression of DLEU2 is negatively associated with inflammatory factors such as *TNF-α*, *IL-1α*, *IL-1β*, *IL-6*, and *NLRP3*. Mechanistically, DLEU2 exerts anti-inflammatory activity by inhibiting the NF-κB signaling pathway. In conclusion, the lncRNA DLEU2 in the intestinal mucosa is dysregulated upon gut inflammation and may act as a diagnostic biomarker and a therapeutic target for UC.

## Introduction

Ulcerative colitis (UC) is an immune-mediated chronic nonspecific inflammatory disease of the colon and rectum, belonging to inflammatory bowel disease (IBD). The etiology and pathogenesis of UC are not fully understood (Russell and Satsangi, 2004; Irving and Gibson, 2008; Tozlu et al., 2021). The incidence of UC in Asia is much lower than that in Europe, but it has increased significantly in China in recent years (Kaplan, 2015; Gearry, 2016; Yang, 2016). Thus, a deeper understanding of its pathogenesis, diagnosis, and treatment is urgently required.

The pathogenesis of UC is affected by many factors, including genetic susceptibility, epithelial barrier defects, immune response disorders, and environmental factors. UC classification is critical for the clinical management of patients due to the heterogeneous and varying disease courses. Several specific genes have been identified as biomarkers which closely related to UC pathogenesis (Lee et al., 2018). Wang et al. found that Aquaporin (AQP) four deficiency alleviates experimental colitis in mice (Wang G. et al., 2019). Su et al. found that AA genotype of HSP70-2 polymorphisms was associated with severe UC, and HSP70-2 polymorphism may be used to predict UC phenotypes (Nam et al., 2007). TNF-α is the major pro-inflammatory factor of intestinal epithelial cell proliferation and apoptosis, and the expression of TNF-α in intestinal mucosa increased in DSS-induced UC mice, suggesting that TNF-α is involved in colonic inflammation (Watson and Hughes, 2012; Billmeier et al., 2016; Abraham et al., 2017; Schreurs et al., 2019; Hu et al., 2020).

Long non-coding RNAs (lncRNAs) are a class of non-coding RNAs (ncRNAs) defined by a transcript length of >200 nucleotides (Ma et al., 2013). LncRNAs have been implicated as potential therapeutic targets in various diseases such as cancers, metabolic diseases, and inflammatory diseases (Winkle et al., 2021). We found that a lncRNA named Deleted in Lymphocytic Leukemia 2 (DLEU2) was up-regulated in the nidus of the intestinal mucosa of UC patients. DLEU2 is located on chromosome 13q14 and was originally identified as a potential tumor regulator gene (Garding et al., 2013). However, the role of lncRNA DLEU2 in UC remains unknown.

In this study, we compared lncRNA levels between the nidus of the intestinal mucosa and adjacent normal mucosa in UC patients by RNA sequencing. And the role of DLEU2 in inflammation was investigated *in vitro*. DLEU2 is identified to be a potential anti-inflammatory lncRNA and inhibits gut inflammation by negatively regulating the NF-κB signaling pathway. The lncRNA DLEU2 is suggested to be a diagnostic biomarker and anti-inflammation target for UC.

## Materials and methods

### Materials

Anti-IL-1β antibody (AF-401) was purchased from R&D systems (Minneapolis, MN). anti-p-p65 antibody (3031), anti-β-actin antibody (4970S), and HRP-conjugated goat-anti-rabbit IgG (7074S) were obtained from Cell Signaling Technology (Beverly, MA). HRP-conjugated donkey-anti-goat IgG antibody (A0181) was purchased from Beyotime (Shanghai, China). 96T Human TNF-α ELISA kit (CHE0019-096) and 96T Human IL-6 ELISA kit (CHE0009-096) were obtained from 4A Biotech Co., Ltd. (Beijing, China). PrimeScript RT Master Mix reagent kit (R122) and SYBR Green Master Mix (Q141) were purchased from Vazyme (Nanjing, China). Phorbol myristic acetate (PMA, P8139) was purchased from Sigma (St. Louis, MO).

### Subjects and samples collection

A total of four UC patients were included in the study who presented typical UC symptoms. The nidus of the intestinal mucosa and adjacent normal mucosa were taken from subjects during colonoscopy, respectively (>100 ng).

### RNA sequencing and data analysis

Total RNA of the mucosa samples was isolated by Trizol reagent (Invitrogen, Carlsbad, CA, United States), and its quantity and purity were analyzed by Bioanalyzer 2100 and RNA 1000 Nano LabChip Kit (Agilent, CA, United States) with a standard of RIN number >7.0, respectively. After the quality check, ribosomal RNA was removed from 5 µg of total RNA by using Ribo-Zero™ rRNA Removal Kit (Illumina, San Diego, United States). The paired-end sequencing was performed on Illumina Novaseq™ 6000 platform. Reads were mapped into the genome of *Homo sapiens* by hisat2 Ver 2.0.4 (Kim et al., 2019), and assembled by stringtie Ver 1.3.4 (Pertea et al., 2016) with default parameters. StringTie was used to perform expression levels for mRNAs and lncRNAs by calculating FPKM (Pertea et al., 2016). The differentially expressed mRNAs and lncRNAs were selected with log2 (fold change) ≥ 1 or log2 (fold change) ≤ −1 and with statistical significance *p* value < 0.05 by R package edgeR or DESeq2 (Love et al., 2014). KEGG enrichment analyses were used to screen the signaling pathways involved.

### Cell culture

The THP-1 human monocytic leukemia cell line was purchased from the American Type Culture Collection. The cells were cultured in RPMI-1640 medium (HyClone; Cytiva) containing 10% fetal bovine serum (FBS; HyClone; Cytiva) and 1% penicillin/streptomycin (Invitrogen; Thermo Fisher Scientific, Inc.) in a humidified atmosphere of 5% CO_2_ at 37°C. The cells were stimulated with 100 nM PMA in RPMI 1640 medium for 72 h.

### Transient transfection and quantification

A DLEU2 expression vector was constructed by IGEbio (Guangzhou, China) using the pcDNA3.1(+) vector. DLEU2 siRNA and negative control siRNA were designed and synthesized by IGEbio (Guangzhou, China). siRNA for DLEU2 was as follows: 5′- AGU​CUA​CGU​UGG​AGG​UAA​A-3′. Transient transfection was performed using Lipofectamine 2000 Reagent (Sigma-Aldrich, United States). The PMA-differentiated THP-1 cells were transfected with siDLEU2, pcDNA-DLEU2, or controls for 8 h, and further incubated for 24 h with LPS (1 μg/ml) stimulation. Total RNA was isolated and purified using Trizol reagent (Invitrogen, Carlsbad, CA, United States) following the manufacturer’s procedure. Using GAPDH as an internal reference, the relative expression levels of TNF-α, IL-1α, IL-1β, IL-6, NLRP3, and DLEU2 were measured by qPCR with 2^−ΔΔCt^ method. Amplification reactions consisted of an initial denaturation at 95°C for 3 min, 40 cycles of denaturation at 95°C for 15 s, annealing at 60°C for 30 s, and extension at 72°C for 30 s. GAPDH was used as an internal control. Primer sequences are shown in Table 1.

**TABLE 1:** Primers used in RT-qPCR.

	Forward (5′–3′ sequence)	Reverse (5′–3′ sequence)
*GAPDH*	GGT​CGG​AGT​CAA​CGG​ATT​TGG	CCA​TGG​GTG​GAA​TCA​TAT​TGG​AAC
*DLEU2*	CGG​GGT​TGG​CTC​TAA​CGA​AT	GTG​GCG​CTA​TAC​TCT​CGG​AC
*TNF-*α	CCT​CTC​TCT​AAT​CAG​CCC​TCT​G	GAG​GAC​CTG​GGA​GTA​GAT​GAG
*IL-1*α	TGG​TAG​TAG​CAA​CCA​ACG​GGA	ACT​TTG​ATT​GAG​GGC​GTC​ATT​C
*IL-1*β	ATG​ATG​GCT​TAT​TAC​AGT​GGC​AA	GTC​GGA​GAT​TCG​TAG​CTG​GA
*IL-6*	ACT​CAC​CTC​TTC​AGA​ACG​AAT​TG	CCA​TCT​TTG​GAA​GGT​TCA​GGT​TG
*NLRP3*	GAT​CTT​CGC​TGC​GAT​CAA​CAG	CGT​GCA​TTA​TCT​GAA​CCC​CAC

### Western blotting

Total proteins were harvested from human macrophage-like cells derived from THP-1. After transfection, cells in each group were washed by pre-cooling PBS, 100 μl cell lysate was added, lysed at 4°C for 30 min, and centrifuged at 12,000 r/min for 10 min at 4°C to extract proteins. Protein concentration was detected by BCA protein concentration assay kit (Invitrogen, United States). The protein gels were transferred to polyvinylidene fluoride (PVDF) membrane by SDS-PAGE reaction of 30 μg total protein per swimming lane. 5% defatted milk powder was sealed for 1 h, and protein primary antibody (1:1000) was added, respectively, and incubated in a shaker for 24 h (4°C). After washing the film with TBST, the film was incubated with a secondary antibody (1:2000) at room temperature for 1 h. TBST was washed and ECL developed. The gray values of the strips were detected by Gel imaging system and Quantity one software.

### Statistical analysis

Data were analyzed using SPSS version 17.0 statistical software (SPSS, Inc.). Measurement data are expressed as the mean ± standard deviation. An unpaired Student’s t-test was employed to examine the difference between the two groups. *p* < 0.05 was considered to indicate a statistically significant difference.

## Results

### DLEU2 is up-regulated in UC

**FIGURE 1:**
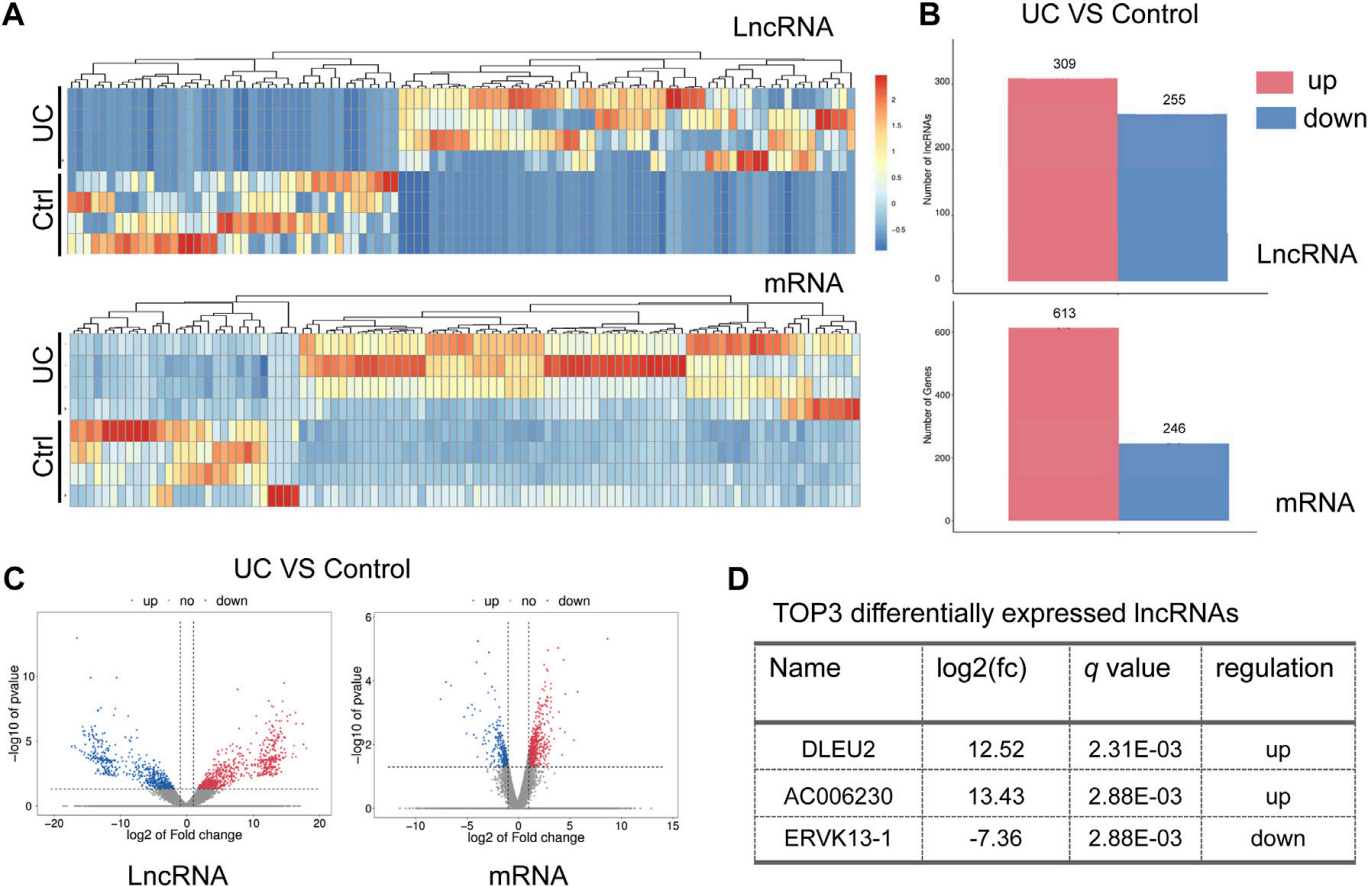
DLEU2 is up-regulated in UC patients. **(A)** The clustering heat map of the differentially expressed lncRNAs and mRNAs. **(B)** The barplot map of the differentially expressed lncRNAs and mRNAs. **(C)** The volcano map of the differential expressed lncRNAs and mRNAs. **(D)** TOP three differentially expressed known lncRNAs. Control, ctrl.

A comparison of RNA expression was made between samples of the nidus of the intestinal mucosa (colitis) and adjacent normal mucosa (control). An overall change of lncRNAs and mRNAs in the intestinal mucosa of UC was observed (Figure 1A). A total of 564 lncRNAs (309 up-regulated and 255 down-regulated) were significantly altered compared to control (Figures 1B,C). And a total of 859 mRNAs (613 up-regulated and 246 down-regulated) were significantly changed (*p* < 0.05) (Figures 1B,C). Of note, the up-regulated DLEU2 changes the most in the differentially expressed (DE) lncRNAs (Figure 1D).

### NF-κB signaling pathway is associated with UC

**FIGURE 2:**
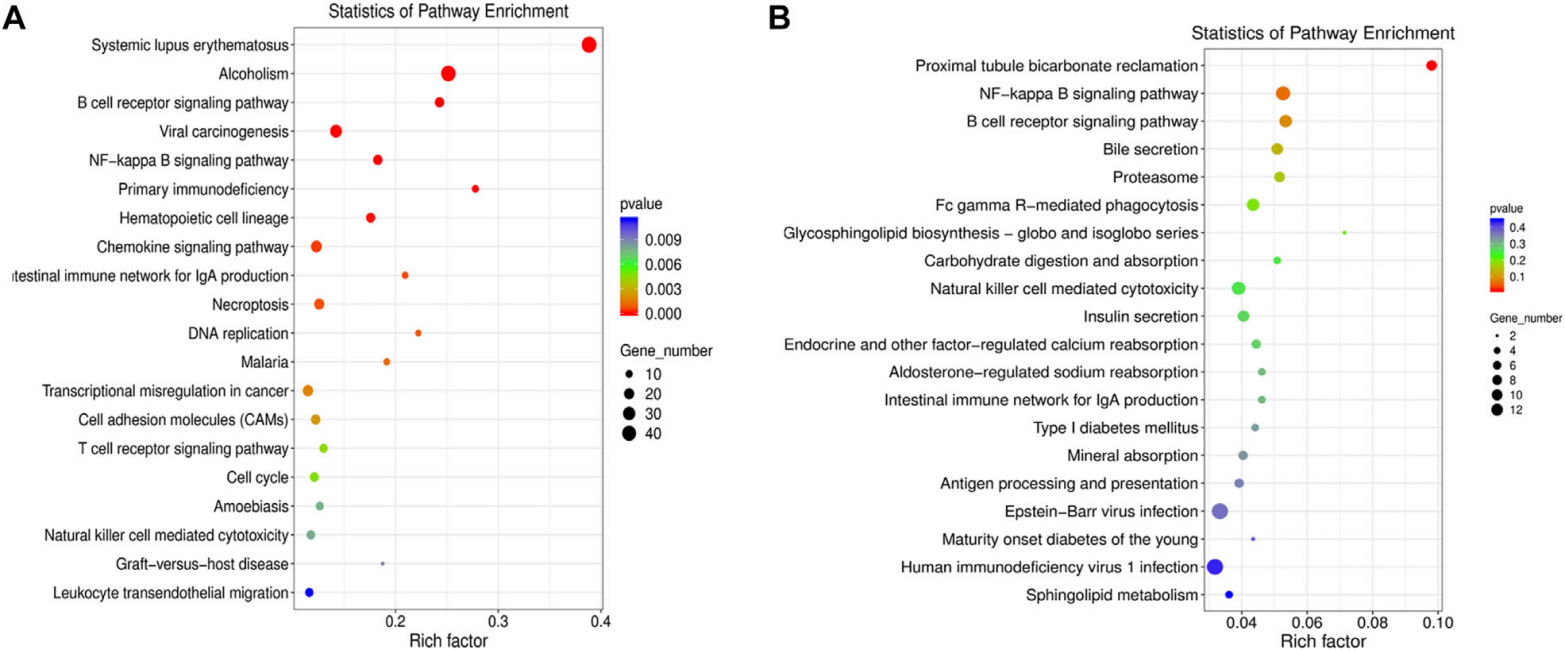
GO and KEGG pathway enrichment analysis of the differentially expressed (DE) mRNAs and DE LncRNAs-targeting genes. **(A)** KEGG pathway enrichment analysis of the DE mRNAs. **(B)** KEGG pathway enrichment analysis of the DE lncRNAs-targeting genes.

The DE mRNAs and lncRNAs were analyzed by KEGG pathway enrichment analyses. The DE mRNAs were mainly enriched in inflammation-related pathways including systemic lupus erythematosus, alcoholism, B cell receptor signaling pathway, viral carcinogenesis, and NF-κB signaling pathway (Figure 2A). We further performed functional enrichment analysis of DE lncRNAs-targeting genes. The DE lncRNA-targeting genes were mainly enriched in proximal tubule bicarbonate reclamation, NF-κB signaling pathway, B cell receptor signaling pathway, bile secretion, and proteasome (Figure 2B). Taking together, the results of enrichment analyses suggested several inflammation-related pathways related to UC. Among them, the signaling pathway NF-κB is closely associated with gut inflammation.

### DLEU2 down-regulated expression of inflammatory genes

**FIGURE 3:**
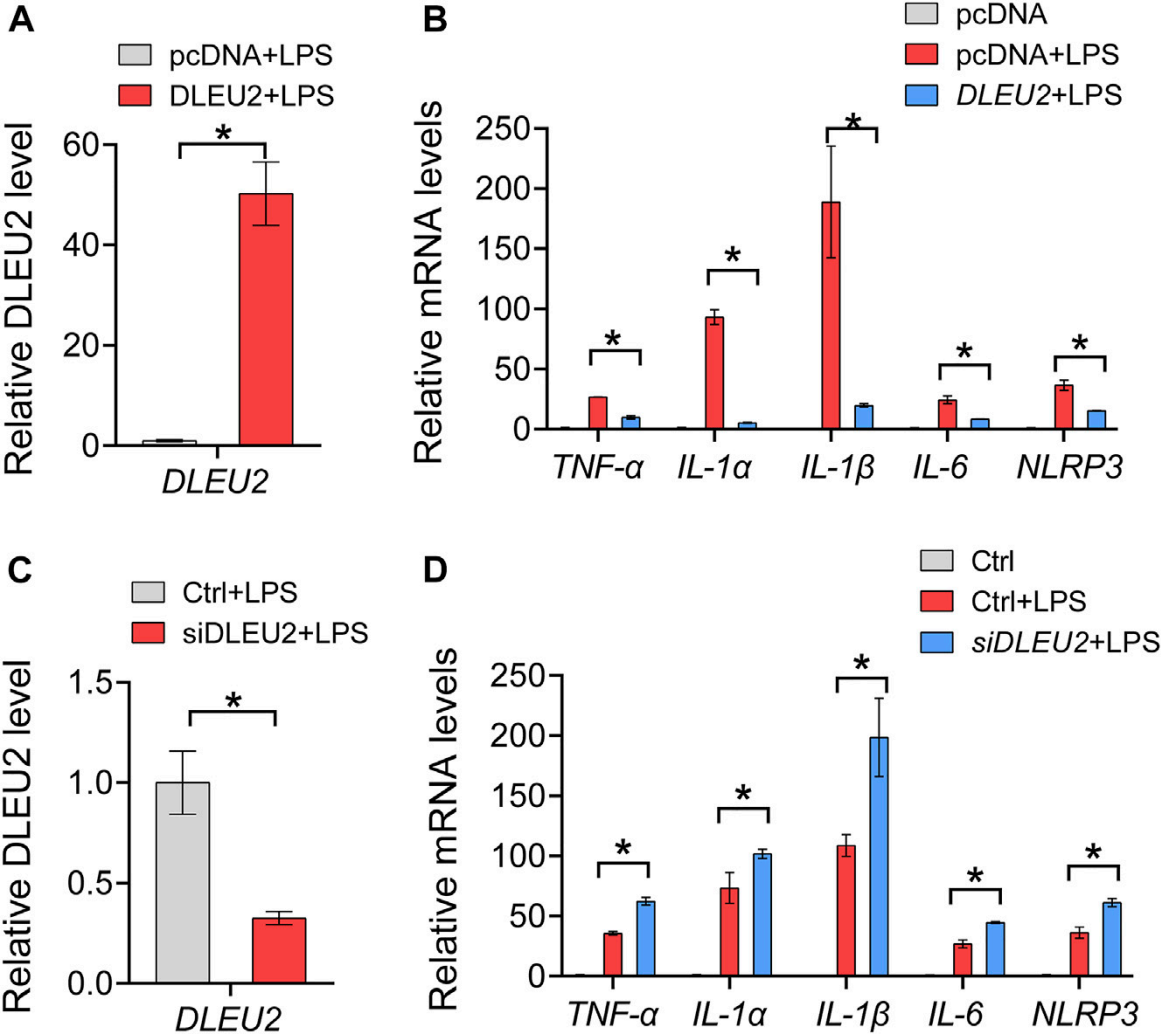
DLEU2 inhibits NF-κB-related inflammation cytokines. **(A)** The expression level of DLEU2 in DLEU2 and pcDNA (control) treated THP-1 cells. **(B)** Effects of DLEU2 overexpression on level of inflammation cytokines in THP-1 cells. **(C)** The expression level of DLEU2 in siDLEU2 and negative control transfected THP-1 cells. **(D)** Effects of DLEU2 knockdown on level of inflammation cytokines in THP-1 cells. *n* = 5 per group. *p* < 0.05 (*t*-test).

We further tested the effects of the DLEU2 on inflammation with macrophage-like cells derived from THP-1. The overexpression and knockdown efficiency of DLEU2 was verified by the RT-qPCR (Figures 3A,C). The expression levels of *TNF-α*, *IL-1α*, *IL-1β*, *IL-6*, and *NLRP3* in THP-1 cells were significantly decreased upon DLEU2 overexpression (Figure 3B). Consistently, these inflammatory factors were significantly increased in the siDLEU2 group compared with the control group (Figure 3D). Overall, DLEU2 inhibits inflammation *in vitro*.

### DLEU2 inhibits NF-κB signaling pathway

**FIGURE 4:**
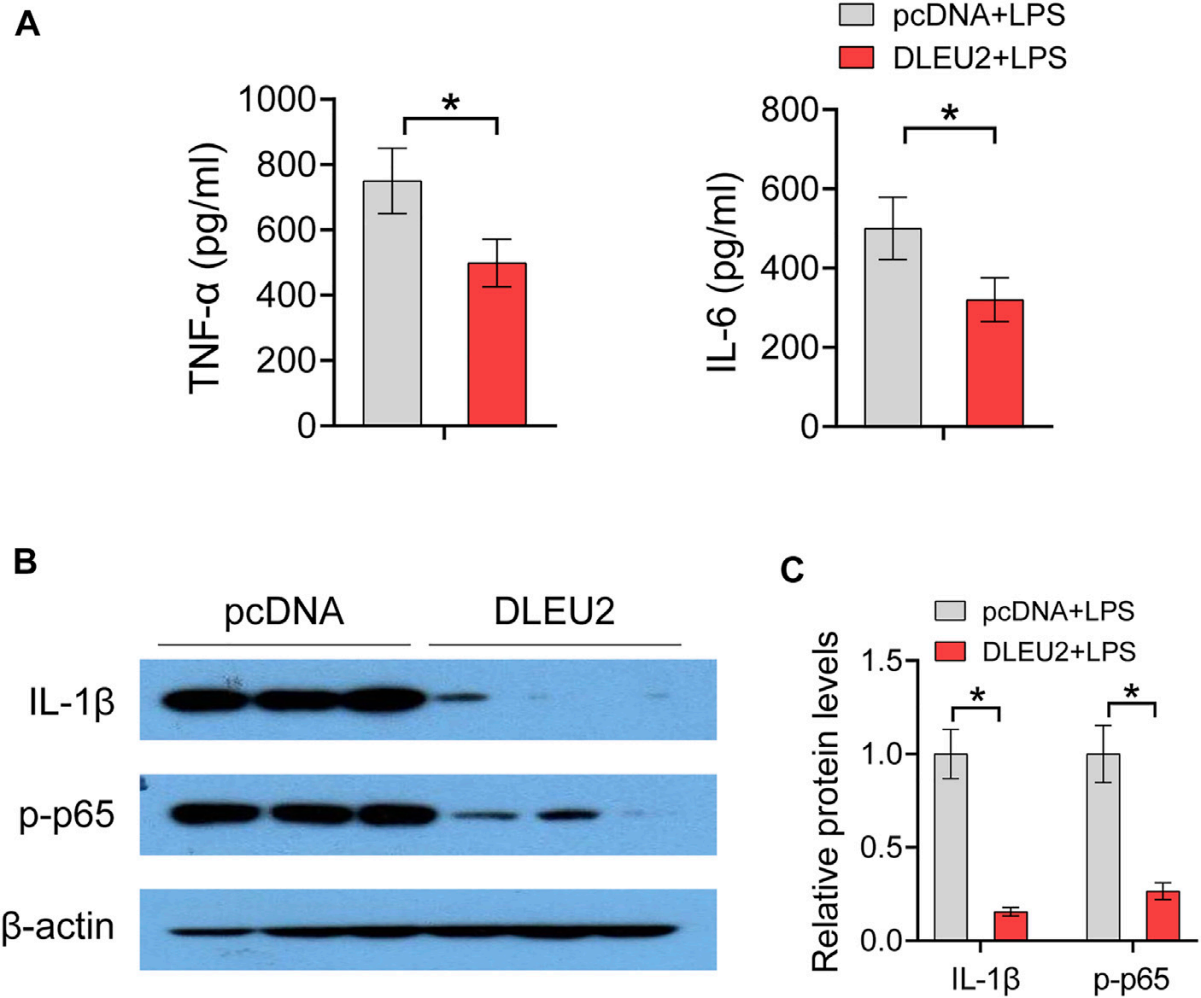
DLEU2 inhibits NF-κB signaling pathway. **(A)** The level of IL-6 and TNF-α protein in THP-1 cells transfected with DLEU2 or pcDNA. **(B)** The protein expression level of IL-1β and p-p65 in THP-1 cells transfected with DLEU2 or pcDNA. **(C)** Quantification data of **(B)**. *n* = 3 per group. *p* < 0.05 (*t*-test).

Of note, the abovementioned inflammatory genes (i.e., *TNF-α*, *IL-1α*, *IL-1β*, *IL-6*, and *NLRP3*) are direct targets of NF-κB. We further investigated the effect of DLEU2 on protein levels of NF-κB-regulated inflammatory factors. DLEU2 significantly decreased levels of TNF-α and IL-6 in LPS-treated THP-1 cells (Figure 4A). In addition, Western blotting assay revealed that the expression levels of IL-1β and phosphorylated NF-κB p65 (p-p65) were also significantly decreased by DLEU2 (Figures 4B,C). Taken together, these results indicate that DLEU2 inactivates the NF-κB signaling pathway to exert anti-inflammatory effects.

## Discussion

In this study, the expression of lncRNAs and mRNAs between the nidus of the intestinal mucosa and adjacent normal mucosa in UC patients were measured and compared. DLEU2 is the most significant DE lncRNA in the intestinal mucosa. DLEU2 was identified as an anti-inflammatory lncRNA. The anti-inflammatory effect of DLEU2 was validated *in vitro*. Mechanistically, DLEU2 inhibits inflammation by negatively regulating the NF-κB signaling pathway, suggesting DLEU2 may serve as a novel biomarker and therapeutic target for UC.

The DE mRNAs and lncRNAs were mainly enriched in NF-κB signaling pathway in UC patients, which indicates the pathological process and inflammatory response of UC are closely associated with NF-κB pathway (Bing et al., 2017; Shen et al., 2019; Tian et al., 2019; Wang L. et al., 2019; Qiu et al., 2020). The interactions of DLEU2 and NF-κB may also be implicated in other physiology and diseases. In previous studies, DLEU2 was often related to cell proliferation and tumor progression. Mikael et al. found that the inactivation of DLEU2 promotes cell proliferation and tumor progression (Lerner et al., 2009). Angela et al. found that DLEU1 and DLEU2 mapped to a critical region at chromosomal band 13q14.3 that is recurrently deleted in solid tumors and hematopoietic malignancies like chronic lymphocytic leukemia. The tumor suppressor mechanism at 13q14.3 is a cluster of genes controlled by two lncRNA genes that are regulated by DNA-methylation and histone modifications and whose members all regulate NF-κB (Garding et al., 2013). Our study reveals that DLEU2 inhibits NF-κB signaling pathway in THP-1 cells. DLEU2 maps at chromosome 13q14.3. The 13q14.3 locus contains C13ORF1, KPNA3, miR-15a/16–1, and RFP2 (Garding et al., 2013). These factors were found to positively regulate NF-κB activity (Garding et al., 2013). These results suggest that DLEU2 may regulate NF-κB through C13ORF1, KPNA3, miR-15a/16–1, and RFP2.

Besides DLEU2, other DE mRNAs and lncRNAs may also play roles in UC. SLC51A gene is involved in bile secretion. Nazzareno et al. found that SLC51, one of the newest members of the solute carrier family, played a critical role in the transport of bile acids, conjugated steroids, and structurally-related molecules across the basolateral membrane of many epithelial cells. In particular, SLC51A appears to be essential for intestinal bile acid absorption, and thus for dietary lipid absorption (Ballatori et al., 2013). Protooncogene TCL1b functions as an Akt kinase co-activator that exhibits oncogenic potency *in vivo* (Hashimoto et al., 2013). We found that TCL1b was highly expressed in the lesion location of the intestinal mucosa, which indicates TCL1b may also relate to the pathological changes in UC.

In conclusion, DLEU2 is identified to be an anti-inflammatory factor and inhibits inflammation by negatively regulating the NF-κB signaling pathway. The lncRNA DLEU2 in the intestinal mucosa is dysregulated upon gut inflammation and may act as a diagnostic biomarker and a therapeutic target for UC.

## Data Availability

RNA-seq data have been deposited into the NCBI SRA database (PRJNA866700). The analyzed data sets generated during the study are available from the corresponding author on reasonable request.
